# Zika Virus Infection in Pregnant Traveler Returning to Denmark from Phuket, Thailand, 2024

**DOI:** 10.3201/eid3102.241510

**Published:** 2025-02

**Authors:** Ingrid Maria Cecilia Rubin, Puk Sandager, Lone Laursen, Shila Mortensen, Vithiagaran Gunalan, Raluca Datcu, Peter H.S. Andersen, Morten Rasmussen, Lasse S. Vestergaard, Uffe Vest Schneider

**Affiliations:** Copenhagen University Hospital, Copenhagen, Denmark (I.M.C. Rubin); Statens Serum Institut, Copenhagen (I.M.C. Rubin, S. Mortensen, V. Gunalan, R. Datcu, P.H.S. Andersen, M. Rasmussen, L.S. Vestergaard, U.V. Schneider); Aarhus University Hospital, Aarhus, Denmark (P. Sandager, L. Laursen); University of Copenhagen, Copenhagen (U.V. Schneider)

**Keywords:** Zika virus, ZIKV, viruses, zoonoses, vector-borne infections, microcephaly, travelers’ health, next generation sequencing, pregnancy, Thailand, Denmark

## Abstract

We report a case of Zika virus (ZIKV) infection in a pregnant woman from Denmark who traveled to Thailand during her first trimester. Late in the second trimester, severe microcephaly was diagnosed in the fetus, and ZIKV infection was confirmed. Travelers and clinicians should be aware of ongoing ZIKV risk in Thailand.

Zika virus (ZIKV) is an RNA virus spread by *Aedes* spp. mosquitoes, and symptoms of ZIKV infection are similar to those of other flaviviruses, such as dengue virus (DENV). A 2015–2016 outbreak in Brazil linked maternal ZIKV infection to serious pregnancy complications, called congenital Zika syndrome (CZS), including miscarriage and birth defects, particularly if the mother is infected during the first trimester (*1*,*2*). Although most ZIKV infections in pregnancy are asymptomatic, the virus can transfer vertically from mother to child, and the estimated risk for transfer is 10%–30% (*3*). Identified in Malaysia in 1966, ZIKV was initially reported in Thailand in 2006, and surveillance that commenced in 2016 confirmed that the virus had been circulating since at least 2002 (*4*).

During March–April 2024, a pregnant woman from Denmark and her partner traveled to Phuket, Thailand, for a 3-week vacation during her first trimester (gestational weeks 8–10) (Figure 1). The couple traveled around Phuket Province and visited the town of Phuket and several popular tourist sites and beaches in the Mueang and Kathu districts. The woman used mosquito repellents but noted a few mosquito bites during her travel. She was not aware of ZIKV risk. On day 12 after her arrival in Phuket, she had mild illness with nausea, loss of appetite, and fatigue for 1 day. Two days later, a maculopapular rash developed on her trunk, arms, and legs.

**Figure 1:**
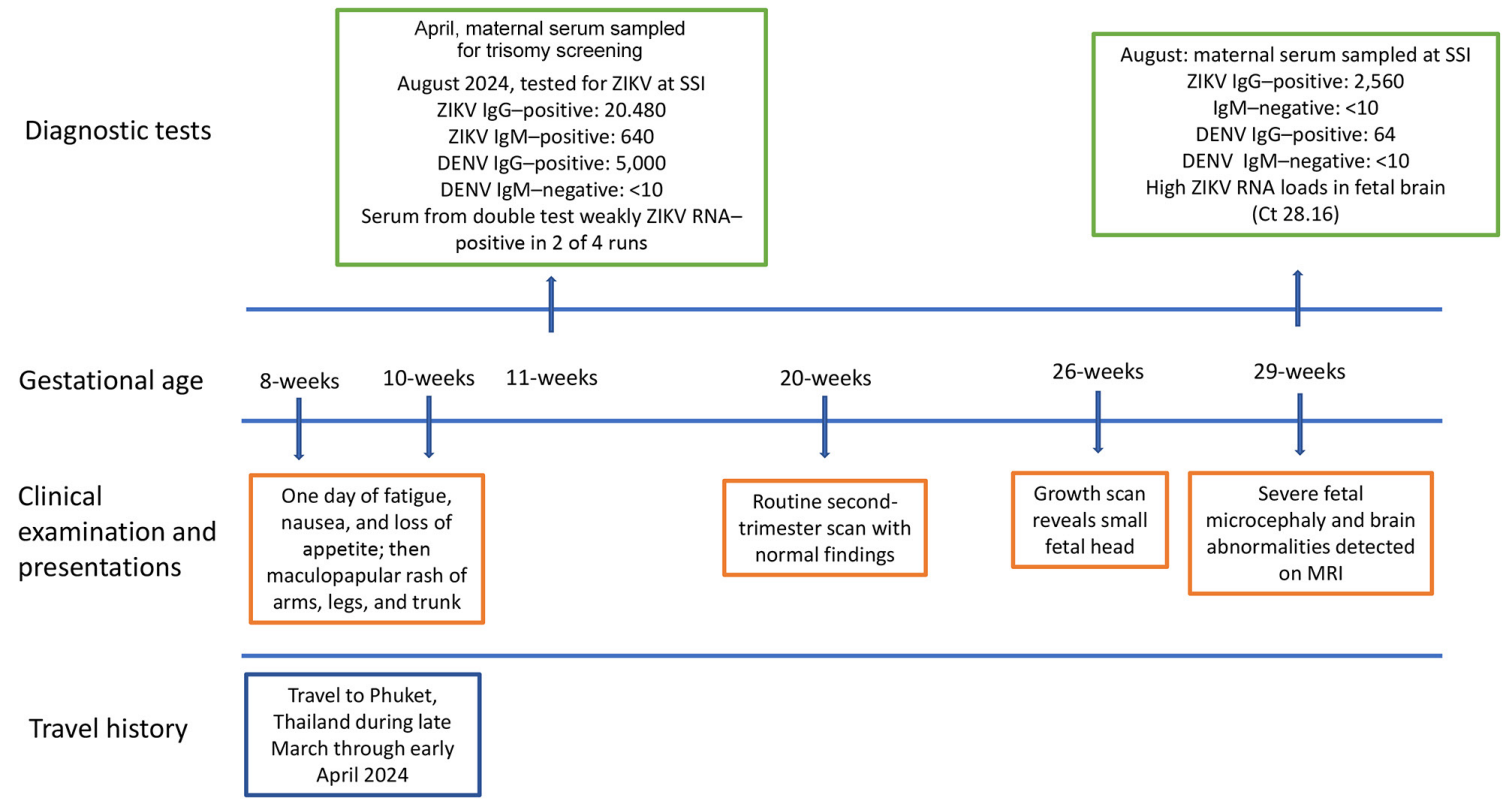
Timeline of patient’s travel history, clinical examinations, and diagnostic tests in case of Zika virus infection in pregnant traveler returning to Denmark from Phuket, Thailand, 2024. Testing was performed the national Virology Reference Laboratory at SSI. Ct, cycle threshold; DENV, dengue virus; MRI, magnetic resonance imaging; SSI, Statens Serum Institut; ZIKV, Zika virus.

Upon her return to Denmark, then in gestational week 11, she had a routine prenatal examination, including collection of a blood sample for trisomy screening, first trimester ultrasound, and combined risk assessment for fetal chromosomal abnormalities. In addition, a chorion villus sample was performed because of fetal chromosomal abnormality in a previous pregnancy. All routine tests showed unremarkable results. The subsequent routine second trimester ultrasound examination also was unremarkable, including appropriate fetal biometrics, and no signs of any anomalies. However, in gestational week 26, an extra growth scan was performed in relation to newly diagnosed gestational diabetes. That scan showed a fetal head circumference substantially below reference size for age (−3.4 SD). 

The patient was referred for further examination. Fetal magnetic resonance imaging revealed severe microcephaly with mild unilateral ventriculomegaly, increased subarachnoid space, and decreased cerebral mantle. Results of serologic testing for toxoplasmosis, cytomegalovirus, and parvovirus were all negative. The pregnancy was terminated because of severe microcephaly.

Serum samples from the woman and fetal tissue biopsy samples were sent to the national Virology Reference Laboratory at Statens Serum Institut (SSI) for ZIKV testing. Reverse transcription PCR (RT-PCR) analysis for ZIKV RNA from the fetal brain showed intermediate positive results, a cycle threshold (Ct) value of 28.16 (Appendix). Formalin fixed samples from the placenta and fetal meninges and liver yielded negative for ZIKV by RT-PCR. Maternal serologic test results from August 2024 showed a high level of ZIKV IgG (titer 2,560) but were IgM-negative for ZIKV. Previous blood samples from gestational week 10 (taken for trisomy screening) showed extremely high ZIKV IgG (20,480) and IgM (640) titers. Furthermore, the sample was positive for DENV IgG with a titer of 5,000 but was DENV IgM-negative. The serum test from the gestational week 10 testing was weakly positive in 2 of 4 replicates, consistent with a weakly RT-PCR positive sample.

Phylogenetic analysis of the consensus genome, designated hZikaV//Denmark/VFU-1/2024 (GISAID accession no. EPI_ISL_19550928; https://www.gisaid.org), showed that the genome clustered with the Asian ZIKV lineage and was closely related to a sequence from a previous case of travel-associated fetal microcephaly from a visitor to Thailand (Figure 2) (*5*). Other genomes observed within this cluster were all sampled from Thailand and Sri Lanka within the previous 8 years. None of the mutations associated with neurovirulence in the other studies were detected (*5*,*6*).

**Figure 2:**
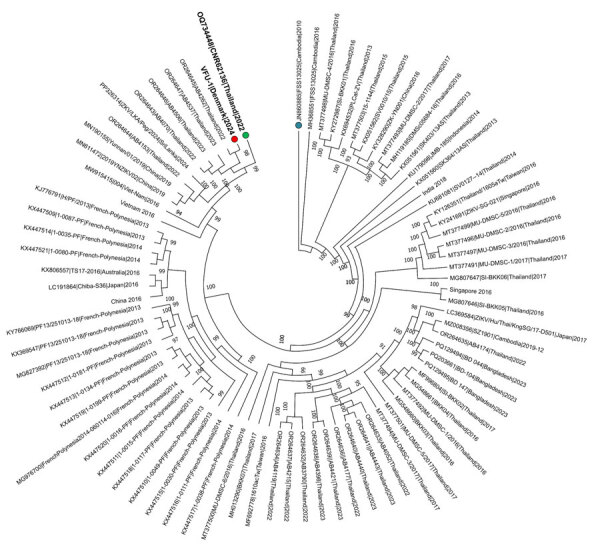
Phylogenetic tree of Zika virus infection in pregnant traveler returning to Denmark from Phuket, Thailand, 2024. Tree was rooted using the oldest genome available in the curated dataset (blue dot); red dot indicates the sequence from this study, VFU-1|Denmark|2024. Green dot indicates a previously sequenced Zika virus genome from a case of travel-associated microcephaly is indicated.

ZIKV is linked to CZS, including microcephaly, especially in symptomatic pregnant women (*3*). However, ZIKV infection often is asymptomatic or causes mild symptoms, as seen in this case. A similar case involved a woman from France who had an asymptomatic travel-related ZIKV infection from Thailand, leading to abortion because of microcephaly detected at 24 weeks’ gestation (*5*).

ZIKV is endemic in 87 countries, and Thailand is a notable source of travel-related infections and CZS cases (*7*,*8*). Diagnosing ZIKV is challenging because of short detection windows and serologic cross-reactivity with other flaviviruses, like DENV (*9*). Furthermore, prenatal microcephaly usually cannot be diagnosed until late in pregnancy.

In this case, ZIKV RNA was strongly detected in the fetal brain and weakly detected in maternal serum. Phylogenetic analysis showed the virus clustered with the Asian lineage circulating in Southeast Asia, which is 1 of the 2 major ZIKV lineages (Figure 2) (*10*).

In conclusion, this case illustrates the ongoing risk for ZIKV infection in Thailand. Obstetricians, travel medicine experts, and other clinicians should recognize the risk for acquired ZIKV infections during travel, and all travelers, especially those who are planning to conceive or are already pregnant, should be aware of the Zika risk and take necessary precautions, such as avoiding travel to ZIKV-endemic countries.

Appendix Additional information on Zika virus infection in pregnant traveler returning to Denmark from Phuket, Thailand, 2024.
