# Supplementary material for: Zika Virus Infection in Pregnant Traveler Returning to Denmark from Phuket, Thailand, 2024
Source: Emerg Infect Dis. 2025 Feb;31(2):382–5. doi: 10.3201/eid3102.241510 (PMC11845159; doi:10.3201/eid3102.241510)
Supplement: Appendix — Additional information on Zika virus infection in pregnant traveler returning to Denmark from Phuket, Thailand, 2024. [file 24-1510-Techapp-s1.pdf]

Article DOI: https://doi.org/10.3201/eid3102.241510

EID cannot ensure accessibility for supplementary materials supplied by authors. Readers who have difficulty accessing supplementary content should contact the authors for assistance.

Zika Virus Infection in Pregnant Traveler Returning to Denmark from Phuket, Thailand, 2024

Appendix

Molecular Methods

Biopsies from fetal cerebrum, meninges, liver, and formalin-fixated placenta were subjected to RNA extraction on a MagNApure96 instrument with the DNA and Viral NA small volume kit (Roche). RT-qPCR was performed using an SSI in-house developed hydrolysis probe assay that targets the ZIKV genome, ZikaF: AGGATGGGAAAAGAAGG, ZikaR: CAGCGTCAATATGCTGTTTT & ZikaP: FAM- TCTGGGGCCTGAACTGGAGA-BHQ1. The PCR reaction contained 5 μL of extracted RNA, 400nM of each primer, 100 nM probe mixed with Sensifast Probe One-step kit (Bioline) in a total volume of 25μl, and amplified on a BioRad CFX96 instrument. Cycling conditions were as follows: 30 min at 48°C RT, 10 min at 95°C, 45 cycles of 15 sec at 95°C and 1 min at 60°C.

Serology testing was performed with anti-Zika Virus ELISA (IgG and IgM) (Euroimmun, Germany) according to manufacturer's instructions.

Viral genome from the brain biopsy was amplified using the multiplex PCR method described by Quick et al., generating overlapping amplicons covering the entire genome (*1*). PCR products were validated on an Agilent TapeStation, pooled, and used to prepare sequencing libraries with the Nextera XT protocol (Illumina). Sequencing was performed on an Illumina MiSeq using 2×150 bp paired-end reads.

References

1. Quick J, Grubaugh ND, Pullan ST, Claro IM, Smith AD, Gangavarapu K, et al. Multiplex PCR method for MinION and Illumina sequencing of Zika and other virus genomes directly from clinical samples. Nat Protoc. 2017;12:1261–76. PubMed https://doi.org/10.1038/nprot.2017.066
